# Assessment of Dyspnea in Critically Ill Patients: A Comparative Analysis of Evaluation Scales

**DOI:** 10.7759/cureus.52751

**Published:** 2024-01-22

**Authors:** Gen Aikawa, Ryota Imanaka, Hideaki Sakuramoto, Chie Hatozaki, Takeshi Unoki, Saiko Okamoto

**Affiliations:** 1 College of Nursing, Ibaraki Christian University, Hitachi, JPN; 2 Department of Nursing, Kyorin University Hospital, Mitaka, JPN; 3 Department of Critical Care and Disaster Nursing, Japanese Red Cross Kyushu International College of Nursing, Munakata, JPN; 4 Department of Nursing, University of Tsukuba Hospital, Tsukuba, JPN; 5 School of Nursing, Sapporo City University, Sapporo, JPN; 6 Department of Nursing, Hitachi General Hospital, Hitachi, JPN

**Keywords:** assessment tool, respiratory distress observation scale, mechanical ventilation, intensive care, dyspnea

## Abstract

Purpose

This study aimed to evaluate the Respiratory Distress Observation Scale (RDOS), Intensive Care RDOS (IC-RDOS), and Mechanical Ventilation RDOS (MV-RDOS) as potential markers of dyspnea in ICU patients by describing their relationship with the Dyspnea Visual Analog Scale (D-VAS).

Materials and methods

A researcher and a trained nurse independently assessed ICU patients simultaneously. One researcher assessed the RDOS (IC/MV-RDOS) and the depth of sedation. An objective evaluation using the observational D-VAS was simultaneously performed by a trained nurse.

Results

The correlation coefficients for each scale were 0.338 for the D-VAS and RDOS, 0.239 for the IC-RDOS, and 0.237 for the MV-RDOS, indicating a low correlation. The prediction of self-reported dyspnea showed that each scale's area under the curve (AUC) as a predictor of D-VAS ≥4 was 0.79 (95% Confidence Interval [CI] 0.71-0.87) for RDOS, 0.77 (95% CI 0.68-0.84) for IC-RDOS, and 0.73 (95% CI 0.64-0.81) for MV-RDOS.

Conclusions

The objective rating scales RDOS, IC-RDOS, and MV-RDOS can predict subjective dyspnea to a certain extent; however, they have limitations in accurately discriminating dyspnea intensity.

## Introduction

Dyspnea is one of the most distressing and serious symptoms in the intensive care unit (ICU) and may be indicative of impending patient death [1]. Approximately 50% of ventilated patients experience dyspnea, which is associated with adverse patient outcomes such as anxiety, prolonged mechanical ventilation, noninvasive ventilation failure, and mortality [2-4]. Therefore, it is important to assess and quantify dyspnea in critically ill patients.

Several tools are available for assessing dyspnea. The Dyspnea Visual Analog Scale (D-VAS), a subjective assessment, is a valid tool for patients who can self-report, but it is not applicable for those who cannot self-report [5]. The Respiratory Distress Observation Scale (RDOS) was developed to objectively assess physical and behavioral signs of dyspnea [6]. Subsequently, tools such as the Intensive Care RDOS (IC-RDOS) for patients admitted to the ICU and the Mechanical Ventilation RDOS (MV-RDOS) for ventilated patients have been developed [7,8]. However, the most suitable tools for assessing dyspnea in ICU patients remain unclear.

This novel study compared the validity of the D-VAS, a subjective dyspnea assessment, with objective assessment tools such as RDOS, IC-RDOS, and MV-RDOS.

## Materials and methods

Patients and setting

This prospective observational study was conducted in a 12-bed general ICU at an 800-bed university hospital from February 2020 to February 2021. This study aimed to evaluate RDOS, IC-RDOS, and MV-RDOS as putative markers of dyspnea in ICU patients by describing their relationship with the D-VAS.

In this study, adult ICU patients who had been receiving ventilation for >24 hours were included. Exclusion criteria included patients aged <20 years, patients predicted to die within 48 hours, those who received mechanical ventilation for ≥24 hours before ICU admission, those who received neuromuscular blocking agents, those who had paralysis/neuromuscular disorders, and those with a history of psychiatric illness and could not understand Japanese.

Ethics review and informed consent

The study was approved by the review committees of the Institutional Review Board of the Study Coordinator Center of Ibaraki Christian University (approval #2019-013) and the Clinical Research Ethics Review Committee of the University of Tsukuba Hospital (approval #R01-184). Informed consent was obtained in writing after explaining the details of the study to the patients or their relatives in accordance with Institution Review Board recommendations.

Data collection

Recorded baseline characteristics included age, sex, BMI, reason for ICU admission, and severity of illness. The severity of illness was calculated using the Acute Physiology and Chronic Health Evaluation II at ICU admission. Depth of sedation was assessed using the Richmond Agitation-Sedation Scale at the time of dyspnea measurement. The use of sedative analgesics and ventilation status were investigated at the time of dyspnea measurement.

The RDOS is an eight-item ordinal scale (heart rate, respiratory rate, restlessness, abdominal paradox, neck muscle use during inspiration, grunting, nasal flaring, facial expression of fear) designed to measure the presence and intensity of respiratory distress in adults [6]. The IC-RDOS evaluates five items, including heart rate, neck muscle use during inspiration, abdominal paradox, facial expression of fear, and supplemental oxygen, which were modified from the RDOS for patients admitted to the ICU [8]. The MV-RDOS is a tool that modifies the IC-RDOS supplemental oxygen item to respiratory rate in patients requiring ventilation [7]. The intensity of dyspnea was assessed by D-VAS (10 cm VAS, from "no respiratory discomfort" to "unbearable respiratory discomfort"); a D-VAS of 4 or greater was indicative of dyspnea [7].

A researcher and a trained nurse were responsible for the evaluation. This pair assessed ICU patients simultaneously and independently. One researcher assessed RDOS (IC/MV-RDOS) and depth of sedation. One trained nurse simultaneously performed objective assessments using the observational D-VAS.

Sample size

Owing to the lack of dyspnea data in the ICU population, formal sample size estimation was not conducted; however, based on a previous study [7], we set the observation point at around 120.

Data analysis

Data were presented as numbers and percentages for qualitative data. For quantitative data, data were presented as means and standard deviations for parametric distribution data and medians and interquartile ranges for nonparametric distribution data.

The association between RDOS, IC-RDOS, MV-RDOS, and D-VAS was examined using Spearman’s rank-order correlations. A correlation coefficient of <0.20 was considered as a "slight, almost negligible correlation," 0.20-0.40 as "low correlation," 0.40-0.70 as "moderate correlation," 0.70-0.90 as "high correlation," and >0.90 as "very high correlation." 

The area under the receiver operating characteristic curve (AUC) was employed to evaluate the predictive ability of RDOS in detecting patients with self-reported dyspnea. The sensitivity and specificity of the various RDOS cutoff points were calculated, and the optimal RDOS cutoff score for our sample was determined using the Youden index. Analysis was performed using STATA version 17.0 statistical software.

## Results

Sample characteristics

**Figure 1:**
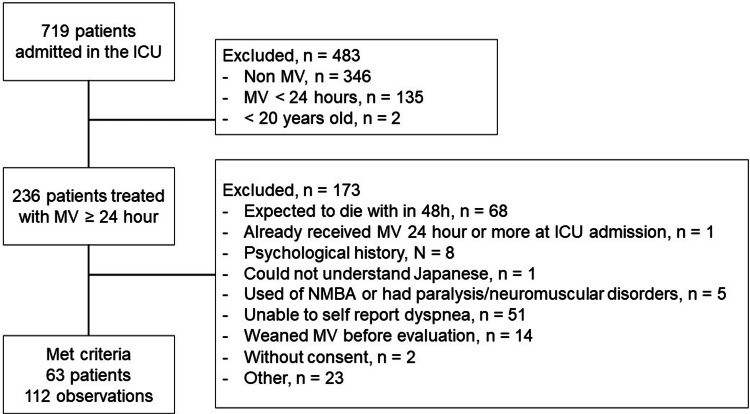
The flowchart depicts the patient recruitment ICU, intensive care unit; MV, mechanical ventilation; NMBA, neuromuscular blocking agent.

In total, 719 patients were admitted to the ICU, and 656 were excluded (Figure 1). Data for the remaining 63 patients were collected, and their characteristics are shown in Table 1. Each participant was evaluated once or twice, and 112 observations were recorded. Self-reported dyspnea was present in 8.0% (9/112) of patients, and the mean D-VAS was 0.98 (1.20).

**Table 1:** Patient characteristics Values are presented as mean ± SD, median (IQR), or n (%). BMI, body mass index; APACHE, Acute Physiology Chronic Health Evaluation; RASS, Richmond Agitation-Sedation Scale; SPONT, spontaneous; PEEP, positive end expiratory pressure; BGA, blood gas analysis; ICU, intensive care unit; SD, standard deviation; IQR, interquartile range.

	Whole Cohort, n = 112	Dyspnea, n = 9	No Dyspnea, n = 103	P-Value
Age, years	67.6 ± 12.8	62.0 ± 13.0	67.9 ± 13.1	0.198
Female	33 (29.5)	3 (33.3)	30 (29.1)	0.791
BMI	24.7 ± 6.0	25.8 ± 5.9	24.6 ± 6.0	0.585
Surgery	79 (70.5)	8 (88.9)	71 (68.9)	0.208
APACHE Ⅱ score	17.3 ± 6.3	15.4 ± 6.9	17.5 ± 6.3	0.350
Emergency admission	43 (38.4)	4 (44.4)	39 (37.9)	0.697
Reason for ICU admission				0.631
Cardiovascular	62 (55.3)	7 (77.8)	55 (53.4)	
Respiratory	9 (8.0)	1 (11.1)	8 (7.8)	
Gastrointestinal	13 (11.6)	1 (11.1)	12 (11.6)	
Neurological	6 (5.4)	0 (0)	6 (5.8)	
Sepsis	5 (4.5)	0 (0)	5 (4.9)	
Others	17 (15.2)	0 (0)	17 (16.5)	
Sedation	42 (37.5)	0 (0)	42 (40.8)	0.015
Analgesia	85 (75.9)	6 (66.7)	79 (76.7)	0.500
RASS	-1 (-1, 0)	-1 (-1, 0)	-1 (-1, 0)	0.083
Ventilation days	6.0 ± 8.2	2.9 ± 1.5	6.3 ± 8.5	0.245
Ventilator setting				
Mode SPONT	46 (41.1)	3 (33.3)	43 (41.7)	0.281
F_IO2_	0.376 ± 0.097	0.408 ± 0.156	0.374 ± 0.093	0.408
PEEP, cmH_2_O	10.7 ± 14.5	6.2 ± 4.7	11.0 ± 14.9	0.342
BGA				
pH	7.42 ± 0.05	7.42 ± 0.04	7.42 ± 0.05	0.689
PaCO_2_, mmHg	39.1 ± 4.3	38.1 ± 4.0	39.2 ± 0.4	0.442
PaO_2_, mmHg	98.3 ± 27.3	85.0 ± 21.7	99.5 ± 27.6	0.130
HCO_3_, mEq/L	24.8 ± 3.2	24.4 ± 1.4	24.8 ± 3.3	0.693
ICU length of stay	10.7 ± 14.5	6.2 ± 4.7	11.0 ± 14.9	0.342
Hospital length of stay	36.1 ± 24.7	25.8 ± 10.9	37.0 ± 25.3	0.193
ICU mortality	6 (5.4)	0 (0)	6 (5.8)	0.457
Hospital mortality	9 (8.0)	0 (0)	9 (8.7)	0.355

Research question results

Regarding the correlation coefficients for each scale, both D-VAS and RDOS were 0.338, the IC-RDOS was 0.239, and the MV-RDOS was 0.237, indicating a low correlation. Furthermore, both the RDOS and IC-RDOS were 0.641, the MV-RDOS was 0.715, and the IC-RDOS and MV-RDOS were 0.901, indicating a high correlation (Table 2).

**Table 2:** Correlations between each scale D-VAS, Dyspnea Visual Analog Scale; RDOS, Respiratory Distress Observation Scale; IC-RDOS, Intensive Care Respiratory Distress Observation Scale; MV-RDOS, Mechanical Ventilation Respiratory Distress Observation Scale.

	D-VAS	RDOS	IC-RDOS	MV-RDOS
D-VAS	1	-	-	-
RDOS	0.338	1	-	-
IC-RDOS	0.239	0.641	1	-
MV-RDOS	0.237	0.715	0.901	1

**Figure 2:**
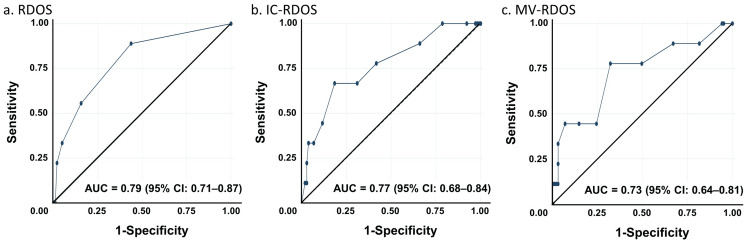
Prediction of self-reported dyspnea by RDOS, IC-RDOS, and MV-RDOS The AUC for RDOS, IC-RDOS, and MV-RDOS as a predictor of D-VAS of 4 or greater. The AUCs were 0.79 (95% CI 0.71–0.87), 0.77 (95% CI 0.68–0.84), 0.73 (95% CI 0.64–0.81), respectively. RDOS, Respiratory Distress Observation Scale; IC-RDOS, Intensive Care Respiratory Distress Observation Scale; MV-RDOS, Mechanical Ventilation Respiratory Distress Observation Scale; AUC, area under the receiver operating characteristic curve; CI, confidence interval.

Prediction of self-reported dyspnea showed that each scale's AUC as a predictor of D-VAS ≥4 was 0.79 (95% confidence interval [CI] 0.71-0.87) for RDOS, 0.77 (95% CI 0.68-0.84) for IC-RDOS, and 0.73 (95% CI 0.64-0.81) for MV-RDOS (Figure 2). The optimal cutoff points and sensitivity and specificity of each scale revealed that RDOS had the highest sensitivity and specificity of 88.9 and 56.3, respectively, when the cutoff was ≥1. IC-RDOS had the highest sensitivity and specificity of 66.7 and 81.6, respectively, when the cutoff was ≥2.5, whereas MV-RDOS had the highest sensitivity and specificity of 77.8 and 68.0, respectively, when the cutoff was ≥2 (Table 3).

**Table 3:** Optimal cutoff points, sensitivity, and specificity of each scale RDOS, Respiratory Distress Observation Scale; IC-RDOS, Intensive Care Respiratory Distress Observation Scale; MV-RDOS, Mechanical Ventilation Respiratory Distress Observation Scale.

	Cutoff Points	Youden Index	Sensitivity	Specificity
RDOS	≥1	0.45	88.9	56.3
IC-RDOS	≥2.5	0.48	66.7	81.6
MV-RDOS	≥2	0.46	77.8	68.0

## Discussion

These results suggest that an objective rating scale may predict dyspnea to a certain degree. However, using an objective rating scale to accurately discriminate dyspnea, which is a subjective symptom, may be difficult. Regarding the correlation between D-VAS and RDOS, the results of previous studies were similar to those of the present study [7]. The correlation between D-VAS and IC/MV-RDOS was low, although it was moderate in previous studies [7,8]. This discrepancy may be attributed to fewer patients (8%) reporting dyspnea in the present study and the resulting differences in analysis methods. Moreover, these findings suggest that higher RDOS, IC-RDOS, and MV-RDOS scores do not necessarily have a linear relationship with the intensity of symptoms accompanying subjective dyspnea.

Moreover, the concurrent validity of the D-VAS and objective rating scale was evaluated. Based on the AUCs of each scale in the present study, the predictive ability of self-reported dyspnea severity was high for the RDOS, IC-RDOS, and MV-RDOS. The AUCs for each measure in this study were first-rate; however, compared to previous studies, the AUCs for RDOS, IC-RDOS, and MV-RDOS were 0.75, 0.86, and 0.77, respectively [7,8], indicating that the predictive ability of IC-RDOS was slightly inferior. This difference may also be attributed to the small sample size of patients who reported dyspnea. No large differences were observed in the AUC for RDOS compared to previous studies. Furthermore, although it was low (0.338), the correlation coefficient was the highest of the three. Therefore, in the ICU setting, the RDOS is the most appropriate because these are the only three tools available to assess dyspnea in patients who cannot self-report.

IC-RDOS and MV-RDOS were highly correlated, the only differences being the oxygen supply and respiratory rates. Therefore, when objectively assessing dyspnea in the ICU, either IC-RDOS or MV-RDOS may be employed regardless of whether or not the patient is on a ventilator. However, the MV-RDOS is composed of respiratory rates, which allow for fine differences in scores and may be versatile for ICU patients.

This study had several limitations. First, this observational study was conducted at a single-center university hospital, and its generalizability was low. Second, to evaluate the correlation with the D-VAS score, patients were assessed after they had recovered sufficiently to be able to self-report. Therefore, the proportion of patients who reported dyspnea was small, which may have influenced our present results. Dyspnea should be assessed under more severe conditions. Third, we set a goal of approximately 120 observations based on the sample size of previous studies, with multiple observations per patient. This could affect the incidence of dyspnea. In the future, a multicenter study will be required to improve sampling efficiency and assure external validity.

## Conclusions

In this study, the objective rating scales RDOS, IC-RDOS, and MV-RDOS were evaluated as putative markers of dyspnea in patients admitted to the ICU by describing their relationship with D-VAS. RDOS, IC-RDOS, and MV-RDOS were found to predict subjective dyspnea to a certain extent; however, it is difficult to discriminate dyspnea accurately. RDOS has the highest correlation with D-VAS of the three tools. Therefore, RDOS may be appropriate when objectively assessing dyspnea in the ICU.
